# Minimizing Enterostomy Complication in Neonates, Lessons Learnt from Three European Tertiary Centres

**DOI:** 10.3390/children9020162

**Published:** 2022-01-27

**Authors:** Riccardo Coletta, Andrea Zulli, Kathryn O’Shea, Elisa Mussi, Adrian Bianchi, Antonino Morabito

**Affiliations:** 1 Department of Paediatric Surgery, Meyer Children’s Hospital, 50139 Florence, Italy; Andrea.zulli@unifi.it (A.Z.); antonino.morabito@meyer.it (A.M.); 2 School of Environment and Life Science, University of Salford, Salford M5 4NT, UK; 3 Department of Neurofarba, University of Florence, Viale Pieraccini 6, 50121 Florence, Italy; 4 Department of Paediatric Surgery, Royal Manchester Children’s Hospital, Manchester M13 9WL, UK; kathryn.m.oshea@gmail.com; 5 Department of Industrial Engineering, University of Florence, 50139 Florence, Italy; elisa.mussi@unifi.it; 6 Royal Manchester Children’s Hospital, Manchester University NHS Foundation Trust, Manchester M13 9WL, UK; bianchi54@gmail.com

**Keywords:** ileostomy, neonates, necrotising enterocolitis, jejunostomy, enterostomy, intestinal atresia

## Abstract

Introduction. Stoma formation in neonates is often a life-saving procedure across a variety of conditions but is still associated with significant morbidity. Tube stoma technique was originally described for short bowel patients, but in selected cases of neonates this approach could prevent the incidence of stoma-related complications. The aim of the study was to evaluate the safety and utility of tube stomas as an alternative to conventional enterostomy in the neonatal population. Material and Methods. A retrospective multicentre analysis of neonates undergoing emergency laparotomy and tube stoma formation between 2005 and 2017 was performed. Tube stoma complications were analysed. The investigation focused on stricture, skin lesion, enteric fistula and prolapse. Results. Thirty-seven neonates underwent tube stoma fashioning during the study period. Tube-stoma complications were limited to three patients (8.1%), with two children (5.4%) requiring additional stoma surgery during the first 30 days because of an enterocutaneous fistula, and one child (2.7%) for bowel stenosis. Conclusions. In select neonates, such as those with proximal enteric stomas, the tube stoma avoids some of the commonly encountered complications (prolapse, skin excoriation). Further prospective studies are needed to validate these findings in order for us to recommend this technique as superior.

## 1. Introduction

The diversionary double-barrel enterostomy commonly used in neonatal and paediatric surgery [1,2,3,4,5,6] carries an appreciable immediate and longer-term morbidity [7,8,9,10,11,12,13,14]. In 2006 Bianchi [15] proposed the ‘tube-stoma’ for controlled bowel expansion and output management for patients with a short bowel. The tube-stoma offers the advantages of not having any exposed bowel with uncontrollable effluent, eliminating contact of irritant fluids with the skin, reducing losses, and improving absorption by retaining nutrients in mucosal contact within the proximal bowel. Manual transfer of collected effluent from the proximal tube-stoma to a separate distal bowel tube-stoma provides an unique opportunity to ‘develop’ the defunctioned distal bowel by stimulating mucosal hyperplasia and increased absorption with reduction in fluid, electrolyte, and nutrient losses [16,17] prior to bowel reconstruction and/or stoma closure. In this paper, we assess the ‘tube-stoma’ as a possible replacement for double-barrel enterostomy in selected circumstances.

## 2. Material and Methods

The Medical Ethical Review Board of our institution stated that this study is based on information routinely collected during normal clinical care, no additional data were collected for the purposes of the study, and no intervention was given solely for the purposes of the study. Therefore, institutional review board approval was waived.

The authors retrospectively examined the clinical case notes of neonates from three European centers who underwent emergency laparotomy and tube-stoma formation between 1 January 2005 and 31 December 2017. Patient demographics and underlying abdominal conditions and stoma complications were analysed according to type and rate of complications and the frequency of surgical revision of the stoma. Categorical variables are presented as counts with percentages (%). Continuous variables are presented as an average value and interquartile range (IQR).

**Figure 1:**
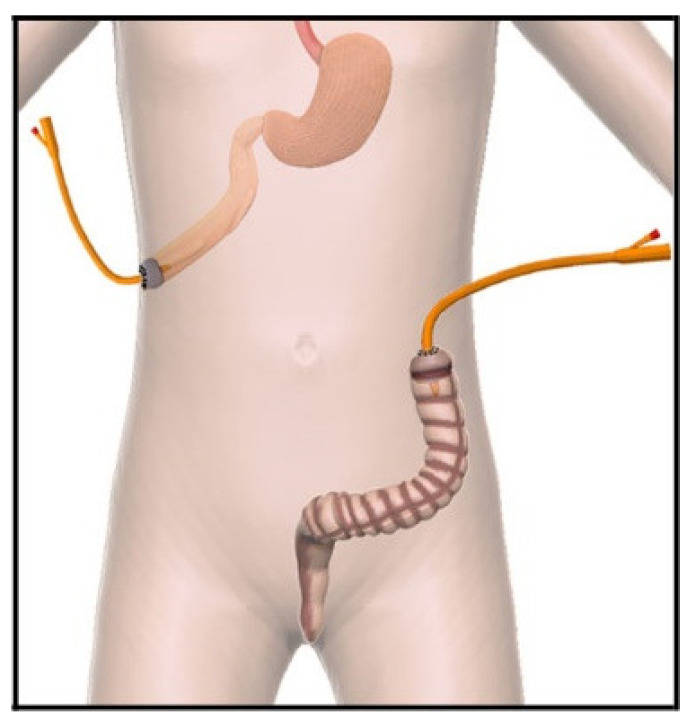
Tube-stoma technique. Representative pictures of tube stoma fashioned in the proximal bowel. In this case, a Foley tube is placed into the lumen of the proximal bowel with the balloon inflated. A double purse-string suture is securing the balloon into the bowel. The same technique is performed in the distal bowel where the tube is placed with the same technique into the lumen of descending colon. The balloon is inflated.

**Figure 2:**
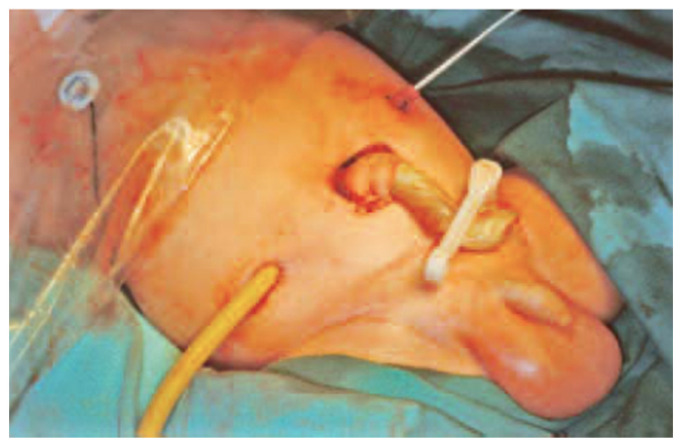
Representative photograph of a patient with tube stoma. The bowel is sutured to the peritoneum at the point of entry of the tube.

Tube-stoma technique [17]: A large tube (size 12–14 Fr Malecot catheter or a 8–10 Fr Foley catheter) is brought through the abdominal wall and passed into the end of the proximal bowel, which is closed around it with a double purse-string suture (Figure 1). The bowel is sutured to the peritoneum at the point of entry of the tube. Through a separate site, a second tube is similarly brought through the abdominal wall and placed in the distal defunctioned bowel (Figure 2). Both tubes are pulled snuggly against the abdominal wall and secured to the skin with tape and/or sutures. Initially the proximal tube-stoma is drained freely but subsequently it is occluded for increasing periods of time, determined by patient tolerance and avoidance of intraluminal infection, to allow for increased absorption from the proximal bowel. Intermittently drained effluent from the proximal tube-stoma is passed slowly, manually, or preferably by syringe-driver during nighttime hours into the distal defunctioned bowel to ‘develop’ the distal bowel mucosa and improve overall fluid and nutrient absorption.

## 3. Results

Over a 12-year period (January 2005 to December 2017), 37 neonates were managed by the tube-stoma method for complications related to abdominal wall defects (43.5%), volvulus (34.8%), necrotizing enterocolitis (13.0%) and intestinal atresia (8.7%). The average birth weight was 2123 g (1850–2735 g) with an average gestational age of 33 weeks (32–35 weeks). All patients underwent bowel resections.

The abdominal surgery and placement of the tube-stomata took place at one to four months of age when the proximal bowel length averaged 25 cm (13–23 cm) from the ligament of Treitz with average diameter 3 cm (2.5–3 cm). Proximal loop output averaged 38 mL/kg/day (32–48 mL/kg/day) with no episodes of tube blockage. A total of 85% of parents manually recycled bowel contents from the proximal to the distal tube, but 15% were minimally compliant.

The tube-stomata were retained for five months (2–6 months) when, at stoma closure, the proximal bowel diameter averaged 5 cm (4–6 cm), with a 72.8% (42.9–100%) increase, and the distal bowel had increased to relatively normal rather than atrophic proportions.

The most frequently reported problem was proximal stoma-tube dislodgment which, for the most part, required simple tube reinsertion. Tube-stoma complications were limited to three patients (8.1%), with two children (5.4%) requiring additional stoma surgery during the first 30 days because of an enterocutaneous fistula, and one child (2.7%) for bowel stenosis (Table 1). No skin complications intended as erosive-ulcerative lesion of the peristomal skin from irritant intestinal content were reported.

## 4. Discussion

A defunctioning enterostomy is not uncommon in neonates, and particularly preterm infants, undergoing emergency laparotomy. Durell et al. found that around 28% of the 16% of neonates born <26 weeks gestation received a stoma. Enterostomy-related complications remain a significant cause of morbidity and occasional mortality with a published rate of 18–100% [3,5,6,7,13,14,18,19,20,21,22,23,24,25,26,27,28,29,30]. The most frequently reported complications are prolapse, retraction, stenosis (7–25%) [5,6,20,25,26,28], necrosis of the stoma, parastomal hernia, enterocutaneous fistula 0–17% [6,7,21,24,29], and breakdown of the skin (4–30%) [7,24,25], while complications such as anastomotic leakage and bowel obstruction may follow stoma closure [7,8,9,10,11,14].

Different studies have sought to identify risk factors for enterostomy-related complications. Lee et al. [29] and Aguayo et al. [25] considered younger gestational age and smaller preoperative weight as significant. Some stoma complications such as strictures have been attributed to ischemic damage to the bowel [31] related to the underlying disease (NEC) rather than to surgical technique. Bishoff et al. [6] stressed an adequate blood supply to the stoma and advocated a large tissue window in all abdominal layers. Strictures that do not adequately respond to dilatation will require stoma revision because of obstruction and bowel dilatation.

High-volume output from small bowel enterostomies may cause significant loss of water and electrolytes [12,13,14]. Ricketts et al. [18] reported that patients with end ileostomies (four patients), but not those with colostomies, had frequent hospital admissions for fluid and electrolyte correction because of diarrhoea, dehydration, and acidosis. Indeed, forced earlier stoma closure may be the only adequate means of controlling fluid and electrolyte balance [29].

Factors such as diet and length of the distal bowel may have an impact on the overall output.

In our patients, managed with the tube-stoma technique, easy proximal-to-distal manual recirculation of bowel content reduced the need for forced early stoma closure. Furthermore, patients practicing bowel content recycling had a better developed distal bowel and a wider, safer anastomosis at stoma closure.

Stoma prolapse is a common enterostomy complication [6] with a frequency of 4 to 26% [3,5,6,7,19,20,24,25,26,29,30]. Responsible factors in surgical technique include an incorrect stoma site (outside the rectus muscle) and undue space between the abdominal wall and the stoma [32]. Some authors affirm that prolapse follows redundant bowel and poor tissue integrity or an wide fascial defect distorted by initial bowel oedema [25]. Prolapse is mostly benign, but major prolapse requires laborious nursing care and may lead to stoma necrosis and significant surgical intervention with loss of precious bowel [6]. Musumeche et al. found similar complication rates for double-barrel stomas and Mikulicz stomas in neonates treated for NEC [31], and Vanamo et al. [33] reported a high complication rate with the Santulli enterostomy for NEC; however, 95% of the neonates were <28 weeks gestational age and had a birth weight of <1000 g.

When comparing our tube enterostomy patients to data from the literature, the conventional stoma techniques have a higher rate of stricture, prolapse, and skin lesions from irritant intestinal content (30%) [34], but no difference for enteric fistula. This is not surprising since tube enterostomy patients do not have any bowel open onto the skin surface so there is no risk of prolapse, and bowel content drains through the proximal enterostomy tube eliminating contact with the abdominal skin. Furthermore, there is no requirement for a stoma bag, which rarely attaches well in small neonates, and can add significantly to skin problems.

Reduction in enterostomy losses and increased absorption are particularly relevant to the child with a short bowel. For these high morbidity patients requiring relatively longer-term management, Bianchi [15,17] proposed controlled, timed occlusion of the tube-stoma, prior to bowel lengthening, to increase nutrient contact time and absorption while avoiding stasis and infection within the loop, and specifically to force a gradual proximal loop dilatation with mucosal hyperplasia and the development of additional small bowel tissue. A major advantage of the tube-stoma is the ability to control and maximize the nutrient-to-mucosa contact time for increased absorption from the proximal bowel, and to avoid stasis and intraluminal infection. Remaining enteric fluids are collected through the proximal tube and manually or syringe-driver recycled in a controlled manner to the defunctioned distal bowel that is thus ‘developed’ in size, texture, and absorptive capability before stoma closure. In our series, we noted a tangible difference in the texture and a wider diameter of the distal bowel when compared with a conventional open stoma. As recycling to the distal bowel increased, there were fewer problems from fluid and electrolyte losses, and a more stable weight gain which was sustained after stoma closure.

The main disadvantage of the tube-stoma is tube dislodgement, which is markedly reduced by careful nursing management during the early postoperative phase. Early replacement of the tube may require radiological guidance but, within some 3–6 weeks once the abdominal tract has matured, the tube can simply be replaced at bedside [17].

Study Limitations: Limitations of the study is that presently few tube-stoma procedures are performed and the rate of complications are still limited. The lack of long-term data decreases the ability to advocate for one approach over another, particularly if the durability of surgical repair is an important factor for the patient.

## 5. Conclusions

Our study is encouraging but is limited by its retrospective nature and lack of data relating to operative time, surgeon experience, and the impact of underlying illness on the complication rate. This study suggests that a proximal tube-stoma avoids some of the commonly encountered complications (prolapse, skin excoriation) associated with conventional enterostomy in neonates. Timed stoma closure increases nutrient-to-mucosa contact time with greater absorption from the proximal loop and has the added advantages of stimulating mucosal hyperplasia while avoiding stasis and bacterial translocation. For the patient with a short bowel, it is invaluable for increasing small bowel surface area and absorption by inducing proximal loop dilatation and mucosal hyperplasia in preparation for bowel lengthening. Controlled drainage of effluent, with the possibility of recycling to the distal loop, further increases absorption and stimulates distal bowel development prior to planned stoma closure or bowel reconstruction.

This paper extends clinical indications for the double tube-stoma to replace open enterostomy for ‘vulnerable’ neonates, thereby reducing complication rates, increasing absorption, and enhancing weight gain. Presently, few tube-stoma are performed and it is only through further application and careful data collection and analysis that the value of tube-stoma relative to the routine open enterostomy can be ascertained.

## Figures and Tables

**Table 1:** Tube stoma complications were limited to three patients (8.1%), with two children (5.4%) requiring additional stoma surgery during the first 30 days because of an enterocutaneous fistula, and one child (2.7%) for bowel stenosis.

Complications	Tube Stoma	
	N. pt	Rate
Enterocutaneous Fistula	2	5.4%
Stricture	1	2.7%
Skin lesions	0	
Prolapse	0	
Total	3	8.1%
